# Noncontrast MRI Assessment of Oxygen Metabolism in Human Calf Muscles Using VSASL and ANN‐Based GESSE

**DOI:** 10.1002/nbm.70387

**Published:** 2026-09-11

**Authors:** Congcong Fu, Hanyi Zhang, Hans‐Ulrich Kauczor, Hyungseok Jang, Sam Sedaghat, Ke Zhang

**Affiliations:** ^1^ Department of Diagnostic and Interventional Radiology Heidelberg University Hospital Heidelberg Germany; ^2^ Translational Lung Research Center (TLRC), German Center for Lung Research (DZL) Heidelberg Germany; ^3^ Department of Diagnostic and Interventional Radiology With Nuclear Medicine Thoraxklinik at Heidelberg University Hospital Heidelberg Germany; ^4^ Department of Radiology University of California Davis Sacramento California USA; ^5^ Division of Radiology German Cancer Research Center Heidelberg Germany

**Keywords:** exercise, magnetic resonance imaging (MRI), oxygenation, perfusion, skeletal muscle

## Abstract

To investigate the feasibility of a noncontrast MRI framework combining velocity‐selective arterial spin labeling (VSASL) and artificial neural network (ANN)–based gradient‐echo sampling of spin‐echo (GESSE) analysis for quantifying exercise‐induced changes in calf muscle perfusion and oxygen metabolism. Ten healthy volunteers (mean age: 31.1 ± 5.45 years) underwent MRI scans before and after standardized eccentric calf muscle exercise. Muscle blood flow (MBF) was assessed using VSASL with 3D GRASE readout, while muscle oxygen extraction fraction (MOEF) was estimated from GESSE data using an ANN‐based method. Skeletal muscle oxygen consumption (SMVO_2_) was calculated based on Fick's principle. All maps were registered to T2‐weighted images, and linear mixed‐effects models were applied to evaluate the effects of time and muscle group. Following exercise, a significant increase in MBF was observed (*Δ* = 6.91 mL/100 g/min, *p* < 0.001), primarily in the gastrocnemius and soleus muscles. MOEF also increased significantly (*Δ* = 0.014, *p* = 0.029), with no significant differences across muscles. SMVO_2_ showed a significant overall increase (*Δ* = 25.56 μmol O_2_/100 g/min, *p* < 0.001), with a significant interaction between time and muscle (*p* = 0.028). The greatest SMVO_2_ increases were observed in the gastrocnemius (68.76% ± 36.1%) and soleus (51.94% ± 44.0%). This noncontrast approach using VSASL and ANN‐based GESSE demonstrates the feasibility of spatially resolved quantification of calf muscle hemodynamics and oxygen metabolism. This method may serve as a valuable tool for assessing microvascular and metabolic function in both research and clinical practice.

## Graphical abstract

Workflow (Steps 1–4): Both MBF and MOEF maps were registered to a T2‐weighted fat‐suppressed structural image using rigid or affine transformations in 3D Slicer. The gastrocnemius, soleus, and tibialis anterior muscles were segmented in 3D volumes, and the resulting segmentations were used as masks to propagate to all parameter maps to extract regional MBF, MOEF, and SMVO_2_ values. SMVO_2_ was calculated from MBF and MOEF using Fick's principle (SMVO_2_ = [*O*_2_] × MOEF × MBF).

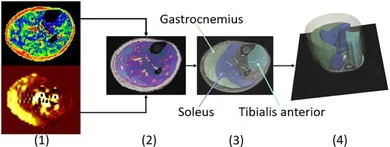

## 1 Introduction

Skeletal muscle oxygenation plays a central role in exercise physiology and serves as a critical biomarker in various pathophysiological conditions, including peripheral artery disease, diabetes, and heart failure [1, 2, 3]. Assessment of skeletal muscle oxygen consumption in patients has yielded inconsistent results, largely due to methodological limitations [1, 4, 5, 6, 7]. Noncontrast MRI mapping of muscle blood flow (MBF) and muscle oxygen extraction fraction (MOEF) enables quantitative assessment of skeletal muscle perfusion and oxygenation [8]. Importantly, skeletal muscle oxygen consumption (SMVO_2_), as an oxygen consumption–related metric, can be quantitatively estimated by combining MBF and MOEF [9]. These parameters provide valuable insights into tissue perfusion, oxygen delivery, and metabolic function, which not only reflect tissue aerobic capacity but also help detect early alterations in microcirculatory function and structure [10, 11].

Arterial spin labeling (ASL) MRI is a noninvasive perfusion imaging technique that enables quantification of tissue blood flow without contrast agents or exposure to ionizing radiation [12]. ASL magnetically labels arterial blood to generate quantitative perfusion maps, with proven value for research and clinical applications in the brain and beyond [13, 14]. However, spatially selective ASL suffers from label decay as the tagged blood travels from large arteries to the microvasculature, often resulting in substantial signal loss and underestimation of perfusion [15]. Velocity selective ASL (VSASL) labels blood based on flow velocity and generates a bolus near the microvasculature [16, 17]. It reduces sensitivity to transit delays, improves labeling efficiency, and provides robust MBF maps suitable for clinical applications [18, 19]. Qin et al. [20] further established a two‐compartment kinetic model for VSASL and demonstrated its accuracy and robustness in brain perfusion mapping.

The discovery of the blood oxygenation level–dependent (BOLD) effect in 1990 [21], followed by the development of an analytical tissue model in 1994 [22], enabled noninvasive mapping of the OEF using MRI, without ionizing radiation and with high spatial resolution. BOLD‐MRI has been verified in both brain and skeletal muscle to assess oxygenation [23, 24], but it generally provides only qualitative information. Gradient‐echo sampling of the spin‐echo (GESSE) has been proposed as a more efficient acquisition strategy [25, 26]. By embedding multiple gradient echoes around a spin‐echo, GESSE simultaneously samples both T2 and T2* signals, facilitating the estimation of OEF‐related parameters within a single scan. However, conventional least‐squares regression (LSR) methods used for quantitative blood oxygen level‐dependent (qBOLD) parameter estimation still require high SNR for stable performance, which limits their application under clinical or time‐constrained conditions [25, 27]. Machine learning methods, including support vector machines and artificial neural networks (ANNs), are increasingly applied in medical imaging processing [28, 29]. Compared with conventional LSR, ANN‐based models offer enhanced robustness to noise and more efficient curve fitting for estimating OEF‐related parameters from GESSE data, making them a promising approach for enhancing the clinical feasibility of OEF mapping [30, 31, 32, 33, 34]. Domsch et al. [35] demonstrated that the ANN approach provides more robust OEF mapping in the human brain of healthy volunteers compared with the conventional LSR method and evaluated the efficiency and accuracy in different ANNs with 5, 10, and 15 hidden layer nodes.

In recent years, noninvasive MRI‐based imaging techniques have obtained increasing attention for investigating skeletal muscle oxygen metabolism. Elder et al. [36] combined BOLD and ASL to quantify changes in oxyhemoglobin saturation (%HbO_2_) during isometric muscle contraction. However, compared to %HbO_2_, SMVO_2_ provides a more direct index of oxygen extraction and metabolism‐related changes. This method enables a noninvasive and noncontrast assessment of tissue oxygen metabolism [8]. A previous study suggested that if MBF and SMVO_2_ can be measured simultaneously, the relationship between muscle oxygen supply and consumption can be more fully characterized [37]. Previous studies have demonstrated the feasibility of deep learning (DL)–assisted OEF mapping in brain MRI [26, 35, 38, 39]. However, applications of combined DL‐assisted perfusion and oxygen metabolism mapping in skeletal muscle remain limited. The novelty of the present study primarily lies in the integration of VSASL and ANN‐based GESSE for noncontrast assessment of skeletal muscle perfusion and oxygen metabolism, rather than in the ANN framework itself.

Taken together, the aim of this study was to evaluate a noncontrast MRI framework combining VSASL and ANN‐based GESSE analysis for regional assessment of skeletal muscle perfusion and oxygen metabolism. This framework may provide technical support for future clinical applications in skeletal muscle disorders and other conditions associated with altered perfusion and oxygen metabolism.

## 2 Methods

### 2.1 Participants

**FIGURE 1:**
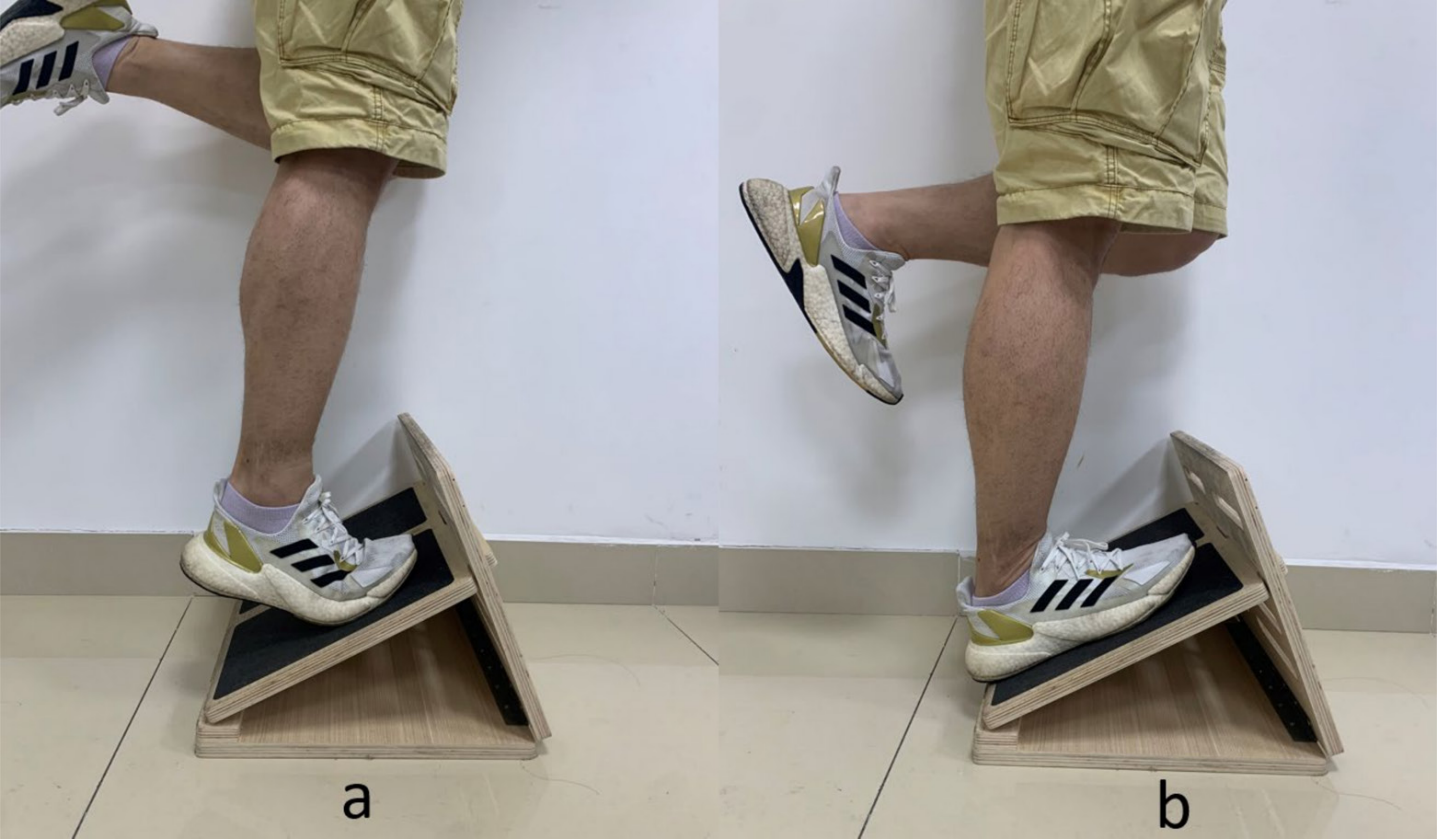
All participants performed a standardized calf muscle EE on a custom‐built slant plate following a warm‐up. They raised one of their heels as high as possible (a), held the position for 1 s, then slowly lowered them over 3 s until the soles touched the bottom (b). Then, return to the starting position was assisted by the opposite leg. Each participant performed four sets of 12 repetitions with 60‐s breaks between sets; the final two sets were continued to the point of fatigue, defined as visible leg tremor and inability to control the descent speed.

This study was approved by the Heidelberg University Institutional Ethics Committee, and all participants provided written informed consent (S‐378/2025). Ten healthy volunteers (six men and four women; mean age, 31.1 ± 5.5 years; mean body mass index, 22.3 kg/m^2^) were included in the study. The inclusion criteria were as follows: no professional training or little experience with weight‐lifting exercises during the 30‐day period before study participation; no known neuromuscular, metabolic, vascular, or musculoskeletal disorders that could potentially affect skeletal muscle perfusion or oxygen metabolism. To minimize confounding effects, participants were instructed to abstain from any form of calf muscle exercise training for 7 days prior to the examination and throughout the study duration. All participants performed a standardized calf muscle eccentric exercise (EE) protocol on a slant plate following a warm‐up [40] (Figure 1). They raised one heel as high as possible (a), held the position for 1 s, and then slowly lowered it over 3 s until the sole contacted the platform (b). The return to the starting position was assisted by the contralateral leg to ensure that the movement phase remained purely eccentric. Each participant performed four sets of 12 repetitions with 60 s rest between sets; the final two sets were continued to volitional fatigue, defined as visible leg tremor and inability to control the lowering speed. To avoid systematic laterality bias, participants were randomly assigned to perform the exercise with either the left or right leg (five participants per side).

### 2.2 MRI Sequences

Imaging was conducted on a 3.0‐T Vida scanner (Siemens Healthineers AG, Erlangen, Germany). A VSASL sequence with a 3D gradient‐ and spin‐echo (GRASE) readout was used. Forty repetitions yielded a complete acquisition in 6 min. Partial‐Fourier sampling (PE/slice = 6/8) was used to shorten echo‐train length and acquisition time. Fat saturation = on (140 mm). Sequence parameters were: TR = 4500 ms, TE ≈ 18.6 ms, flip angle = 120°, shots = 2, FOV ≈ 220 × 220 × 120 mm^3^, matrix size = 64 × 64 × 24, resolution = 3.44 × 3.44 × 5 mm^3^; bandwidth ≈ 2056 Hz/px. For VSASL: labeling/control duration = 64 ms, cutoff velocity = 2.8 cm/s, A Fourier transform–based velocity‐selective inversion pulse train was used. For sinc‐modulated Velocity Selective Inversion (sinc‐VSI), the amplitude of the *n* ^th^ pulse was given by sinc (*n*/5), where *n* = −4:1:4. The flip angles of these nine rectangular pulses were designed to add up to 180°. The sinc‐VSI was played without VS gradients during control conditions, but with VS gradients along all three directions during labeling. The advantage of this setting is that it shows good resistance to *B*_0_ and *B*_1_ inhomogeneity comparing to FT‐VSI [18]. Delay between the presaturation and the labeling modules = 2 s, postlabeling delay (PLD) = 1200 ms, TR = 6 s, timing for background suppression (BGS) = 520, 910, and 1080 ms. Additionally, an *M*_0_ map without any preparation was acquired.

GESSE was acquired in 3 min. Specific parameters were as follows: FOV = 200 × 200 × 98 mm^3^, matrix size = 100 × 100 × 25, slice thickness = 3 mm, distance factor = 30%, TE = 34 ms, TR = 100 ms, number of total echoes = 64, center echo index = 18, bandwidth = 1429 Hz/px, fat‐saturation = on; echo spacing *Δ*TE = 760 μs.

### 2.3 Data Processing

For VSASL evaluations, MBF was calculated by [20, 39]:

(1) $$\mathrm{MBF}=\frac{6000\cdot \lambda \cdot \Delta M\cdot \exp^{\left(\mathrm{TI}/T_{1b}\right)}}{2\alpha \cdot \tau \cdot M_{0}\cdot SI_{B}},$$

(2) $${\mathrm{SI}}_{B}=1-\exp^{\left(-\frac{T_{\mathrm{sat}}}{T_{1b}}\right)},$$

where *ΔM* is the difference between control and label images; *M*_0_ is the equilibrium magnetization acquired without saturation and labeling at matched TR; *α* is the tagging efficiency (*α* = 0.95) [41]; *TI* is the time between labeling pulse and readout (1.2 s); *T*_1b_ = 1.65 s, as the longitudinal blood relaxation rate [42]; *λ* is the blood/tissue water partition coefficient (0.9 mL/g) [43]; *τ* is the bolus duration (1.176 s); and *T*_sat_ is the saturation duration (2 s). Bias field correction was performed on the spin‐echo center image (echo index 18) using the N4ITK algorithm implemented in SimpleITK. An Otsu‐based automatic tissue mask was applied to exclude background noise during bias field estimation. The estimated bias field was subsequently applied to correct all 64 gradient echoes prior to ANN‐based parameter estimation [44].

For MOEF, a feedforward ANN was employed to analyze GESSE parameters [35]. The network architecture consisted of a 64‐dimensional input layer, two hidden layers with 32 and 10 neurons, respectively (using tansig activation functions), and a four‐dimensional output layer with linear activation. The tansig activation function is a hyperbolic tangent sigmoid function commonly used in neural networks to map input values into the range between −1 and 1. The ANN was implemented using the Neural Network Toolbox in MATLAB. The ANN framework was adapted from the previously reported qBOLD‐based approach proposed by Domsch et al. [35] and followed a progressive dimensionality reduction design for nonlinear parameter regression.

A total of 262,200 simulated samples were generated from a qBOLD model by simulating artificial MR signals across broad predefined ranges of R2, λ, OEF, and signal intensity scaling (Sse). Here, R2 denotes the spin–spin relaxation rate, Sse denotes the static spin‐echo signal amplitude, and *λ* represents the deoxygenated blood volume fraction, a physiologically variable vascular parameter that is distinct from the blood/tissue water partition coefficient used in the MBF calculation. The parameter ranges were intentionally selected to cover a wide spectrum of expected qBOLD signal variations rather than to represent a narrowly defined physiological condition. Specifically, the simulated training dataset consisted of all combinations of OEF = 0.05–1.00 in steps of 0.05, R2 = 2–60 s^−1^ in steps of 2 s^−1^, Sse = 50–600 arbitrary units in steps of 25, and *λ* = 0.10–1.00 in steps of 0.05. Uniform random noise with an amplitude up to 10% of Sse was added to each simulated signal to improve robustness against noise‐related fitting variability. The ANN output parameters were ordered as OEF, R2, Sse, and *λ*. The simulated reference values of these parameters were used as labels. The network was trained using the Levenberg–Marquardt backpropagation algorithm (trainlm), with mean squared error (MSE) as the loss function. Inputs and outputs were normalized to the range [0, 1], and the dataset was split into training, validation, and test sets at a 70:15:15 ratio.

The approach to measuring SMVO_2_ is based on Fick's principle [8, 30]:

(3) $${\text{SMVO}}_{2}=\left[O_{2}\right]\times \text{MOEF}\times \mathrm{MBF}$$

where the constant [O_2_] is the total oxygen content of arterial blood (assumed = 7.99 μmol mL^−1^), as previously described [30].

**FIGURE 2:**
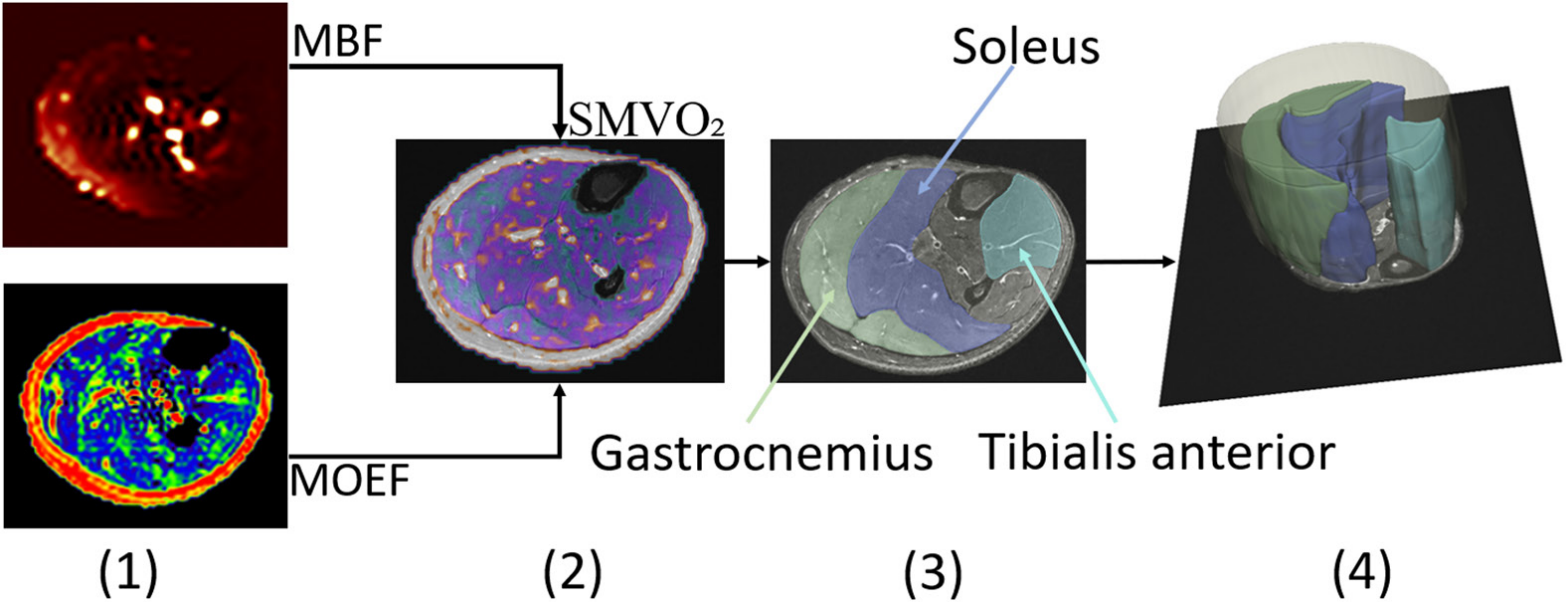
Workflow (Steps 1–4): both MBF and MOEF maps were registered to a T2‐weighted fat‐suppressed structural image using rigid transformations in 3D Slicer. The gastrocnemius, soleus, and tibialis anterior muscles were segmented in 3D volumes, and the resulting segmentations were used as masks that were propagate to all parameter maps to extract regional MBF, MOEF, and SMVO_2_ values. SMVO_2_ was calculated from MBF and MOEF using Fick's principle (SMVO_2_ = [O_2_] × MOEF × MBF). All parameter maps and analyses were performed using custom scripts developed in MATLAB 2024a.

The workflow of the data processing pipeline is illustrated in Figure 2. Both 3D Slicer and MATLAB 2024a were used for data processing, including registration, segmentation, and quantitative analysis. To ensure spatial correspondence between pre‐ and post‐exercise scans, the midbelly of the gastrocnemius muscle was marked on the skin using a surgical marker prior to the first scan session. Upon returning to the scanner after exercise, laser positioning lines were aligned to these anatomical landmarks to reproduce the slice geometry. Within each session, identical slice prescriptions were copied across all sequences to maintain consistent coverage. All parametric maps were subsequently registered to a T2‐weighted fat‐suppressed structural image (fixed reference) using the General Registration (BRAINS) module in 3D Slicer (version 5.1), applying a rigid registration with 7 degrees of freedom (Rigid+Scale, 7 DOF) to correct for any residual positional differences between acquisitions. Muscle regions of interest (ROIs), including the gastrocnemius, soleus, and tibialis anterior muscles, were manually segmented on T2‐weighted images using 3D Slicer. Initial segmentation was performed by a musculoskeletal radiologist with 14 years of experience and subsequently reviewed and confirmed by a second musculoskeletal radiologist with 15 years of experience. The resulting ROIs were then applied to the co‐registered parametric maps for quantitative analysis. MOEF, MBF, and SMVO_2_ maps were computed using custom scripts in MATLAB 2024a (MathWorks, Natick, MA).

### 2.4 Statistical Analysis

All statistical analyses were performed using MATLAB. Data are reported as mean ± standard deviation. Differences in baseline MBF, MOEF, and SMVO_2_ across the three muscles were assessed using a one‐way repeated‐measures ANOVA. Sphericity was examined using Mauchly's test, and Greenhouse–Geisser correction was applied where appropriate. Bonferroni‐adjusted pairwise comparisons were used for post hoc testing. A Friedman test was performed as a nonparametric confirmation. A linear mixed‐effects model (LMM) was used to assess the main effects of time (pre vs. post), muscle group, and their interaction, with subject included as a random intercept. If no significant interaction was found, a simplified LMM model excluding the interaction term was used to estimate the average time effect across muscles. If a significant interaction was observed, post hoc paired *t*‐tests were performed for each muscle group separately. Statistical significance was set at *p* < 0.05.

## 3 Results

**FIGURE 3:**
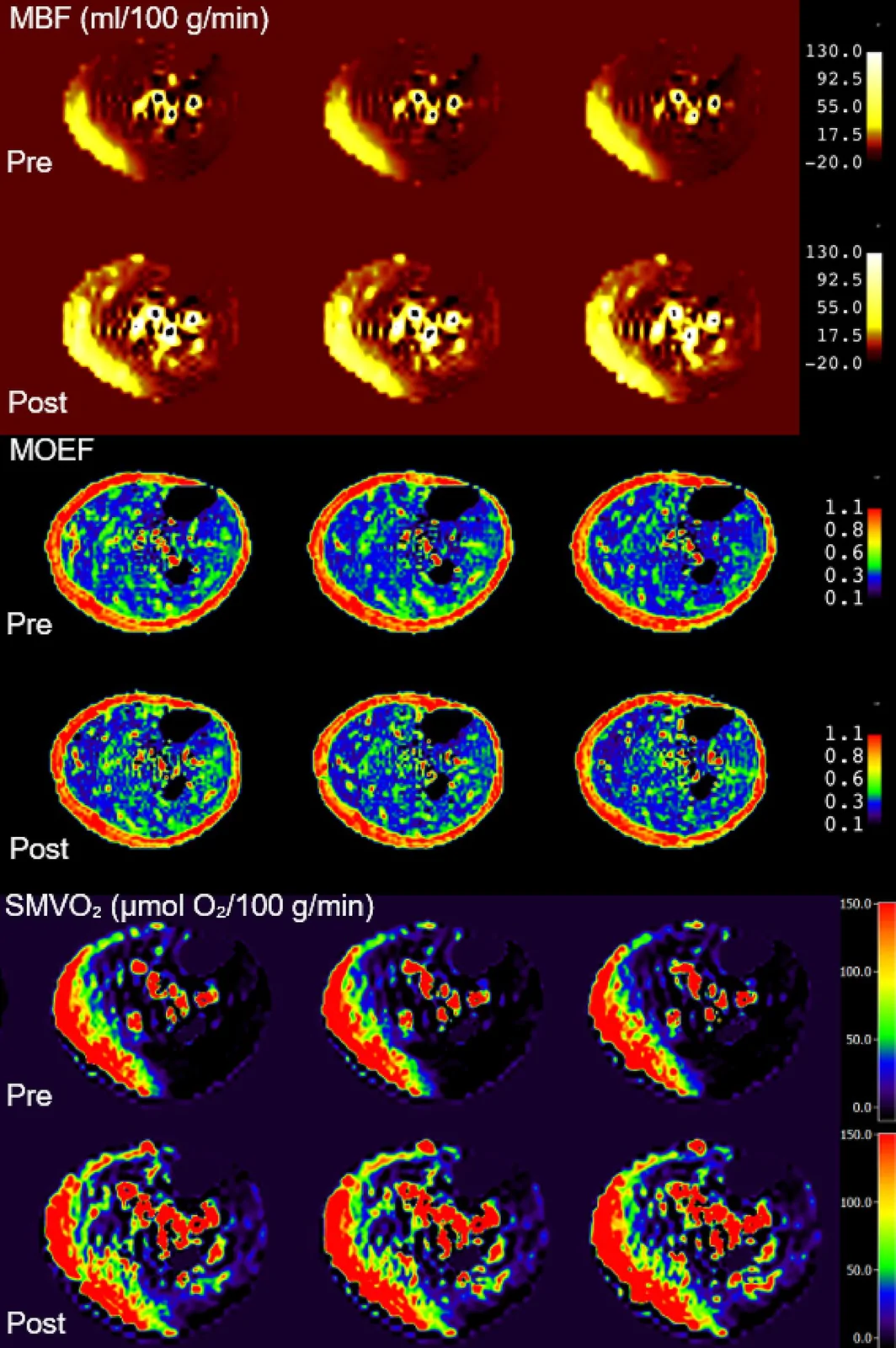
Three consecutive images of the midbelly of the gastrocnemius muscle at two time points before and after EE were selected to illustrate changes in MBF, MOEF, and SMVO_2_. Before EE, MBF in the gastrocnemius muscle was significantly higher than in the soleus and tibialis anterior muscle regions. After EE, MBF increased across multiple regions, with the gastrocnemius showing the most prominent elevation. Before EE, MOEF values across different muscles were relatively low and uneven. After EE, MOEF increased slightly across muscles. For SMVO_2_, oxygen consumption in the gastrocnemius muscle was higher than in the other muscles at rest. After EE, SMVO_2_ increased across all muscles, with the most prominent rise observed in the gastrocnemius. Color bars: MBF is shown in mL/100 g/min (from −20 to 130), MOEF is displayed on a fractional scale (from 0.1 to 1.1), and SMVO_2_ is shown in μmol O_2_/100 g/min (from 0 to 150). Warmer colors represent higher physiological values in all maps.

All data of calf muscles before and after exercise were demonstrated in Tables 1, 2, 3. Representative MBF, MOEF, and SMVO_2_ maps are shown in Figure 3, while the EE protocol and image‐processing workflow are illustrated in Figures 1 and 2, respectively.

**TABLE 1:** Summary of MBF of calf muscles before and after exercise (mean ± SD; mL/100 g/min).

Muscles	Pre‐EE	Post‐EE	*Δ*MBF (mean ± SD)	*p* value	Relative increase (%)
Gastrocnemius	21.52 ± 5.20	34.18 ± 7.91	+12.66 ± 7.28	0.0004	60.44% ± 35.56%
Soleus	9.84 ± 4.22	13.50 ± 5.49	+3.66 ± 3.69	0.0119	47.36% ± 44.22%
Tibialis anterior	12.11 ± 4.96	13.64 ± 7.46	+4.42 ± 9.12	0.1597	38.97% ± 83.61%
Overall	14.49 ± 6.93	21.40 ± 7.86	+6.91 ± 7.98	0.0006 (LMM)	48.92% ± 57.00%

**TABLE 2:** Summary of MOEF of calf muscles before and after exercise (mean ± SD).

Muscles	Pre‐EE	Post‐EE	*Δ*MOEF (mean ± SD)	*p* value	Relative increase (%)
Gastrocnemius	0.419 ± 0.028	0.440 ± 0.026	+0.021 ± 0.036	0.083	5.58% ± 9.58%
Soleus	0.436 ± 0.036	0.449 ± 0.026	+0.013 ± 0.039	0.2395	3.45% ± 8.21%
Tibialis anterior	0.391 ± 0.028	0.399 ± 0.026	+0.008 ± 0.042	0.6038	2.78% ± 12.70%
Overall	0.415 ± 0.035	0.430 ± 0.033	+0.014 ± 0.038	0.0294 (LMM)	3.93% ± 10.05%

**TABLE 3:** Summary of SMVO_2_ of calf muscles before and after exercise (mean ± SD; μmol O_2_/100 g/min).

Muscles	Pre‐EE	Post‐EE	SMVO_2_ (mean ± SD)	*p* value	Relative increase (%)
Gastrocnemius	72.38 ± 19.12	119.67 ± 24.66	47.29 ± 23.84	0.0001	68.76% ± 36.10%
Soleus	34.14 ± 14.98	48.31 ± 17.89	14.17 ± 14.96	0.0151	51.94% ± 44.02%
Tibialis anterior	37.95 ± 16.16	53.16 ± 22.28	15.21 ± 30.56	0.15	41.54% ± 84.52%
Overall	48.16 ± 23.88	73.71 ± 43.98	25.56 ± 16.94	0.0002 (LMM)	54.08% ± 57.90%

At baseline, MBF differed significantly among the three calf muscles. One‐way repeated‐measures ANOVA revealed a significant main effect of muscle type (*F*(2, 18) = 19.56, *p* < 0.001). Mauchly's test indicated a violation of sphericity (W = 0.317, *p* = 0.010); therefore, the Greenhouse–Geisser correction was applied, which confirmed the significant effect (*p* < 0.001). Post hoc Bonferroni‐corrected comparisons showed that the gastrocnemius muscle exhibited the highest MBF (21.52 ± 5.20 mL/100 g/min), significantly higher than the soleus (9.84 ± 4.22 mL/100 g/min, *p* = 0.0013) and tibialis anterior (12.11 ± 4.96 mL/100 g/min, *p* = 0.014). No significant difference was observed between the soleus and tibialis anterior (*p* = 0.102). Representative perfusion maps demonstrated clear spatial differences in perfusion among the calf muscles at rest (Figure 3).

**FIGURE 4:**
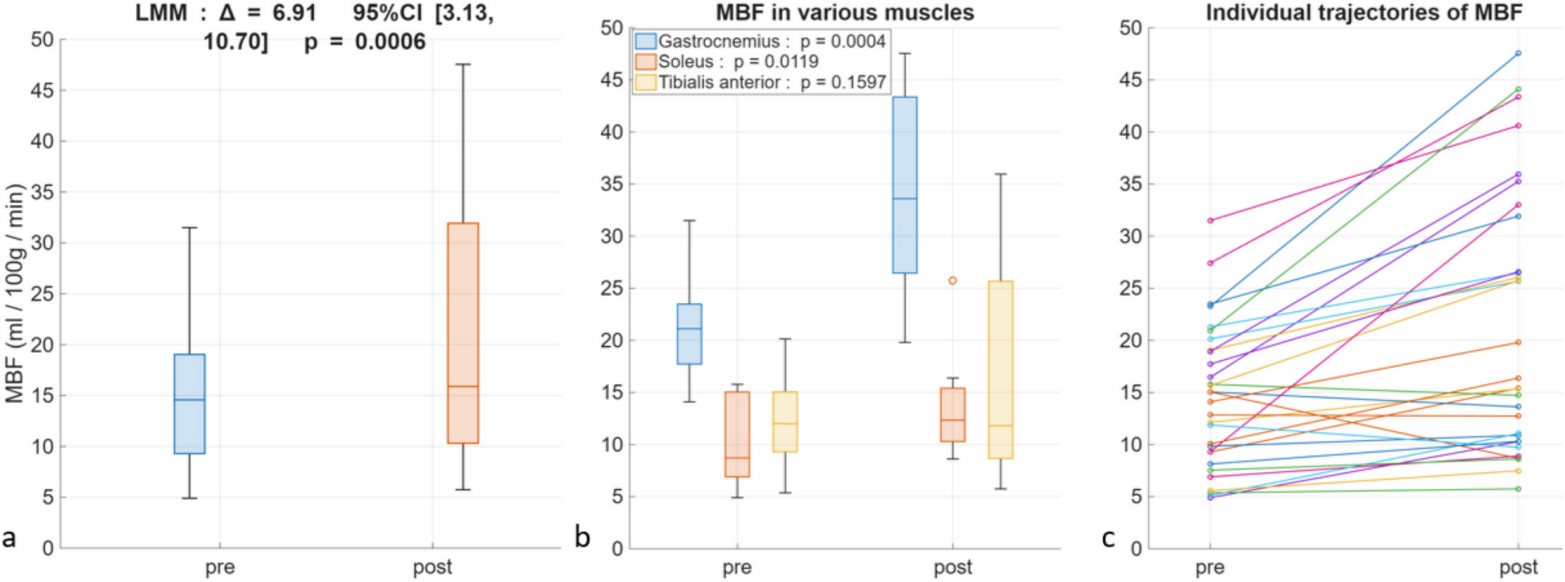
(a) Overall MBF increased significantly after exercise (*Δ* = 6.91, 95% CI [3.13, 10.70], *p* = 0.0006). LMM showed a trend‐level interaction between time and muscle (*p* = 0.074), suggesting possible muscle‐specific effects. (b) The Boxplot shows MBF changes across muscles, with significant increases in the gastrocnemius (*p* < 0.001) and soleus (*p* = 0.012), but not in the tibialis anterior (*p* = 0.160). (c) The Spaghetti plot illustrates consistent post‐exercise MBF increases in most subjects; each line represents the trajectory of an individual subject.

LMM revealed a significant main effect of time (*p* < 0.001), indicating increased perfusion post‐exercise. The interaction between time and muscle was not statistically significant (*p* = 0.074), suggesting a broadly consistent response across muscles. A simplified LMM (excluding the interaction term) estimated an average MBF increase of *Δ* = 6.91 mL/100 g/min (95% CI [3.13, 10.70], *p* < 0.001). Exploratory paired comparisons suggested larger post‐exercise MBF increases in the gastrocnemius and soleus muscles, whereas no clear increase was observed in the tibialis anterior muscle (Figure 4).

At baseline, MOEF differed significantly among the three calf muscles. One‐way repeated‐measures ANOVA showed a significant main effect of muscle type (*F*(2, 18) = 16.56, *p* < 0.001). Mauchly's test indicated that the sphericity assumption was not violated (W = 0.647, *p* = 0.176), and therefore no correction was required. Post hoc Bonferroni‐corrected comparisons revealed that the soleus exhibited significantly higher MOEF (0.436 ± 0.036) compared with the tibialis anterior (0.391 ± 0.028, *p* < 0.001). The gastrocnemius also showed higher MOEF (0.419 ± 0.028) than the tibialis anterior (0.391 ± 0.028, *p* = 0.021). However, no significant difference was observed between the gastrocnemius and soleus muscles (*p* = 0.331; Figure 3).

**FIGURE 5:**
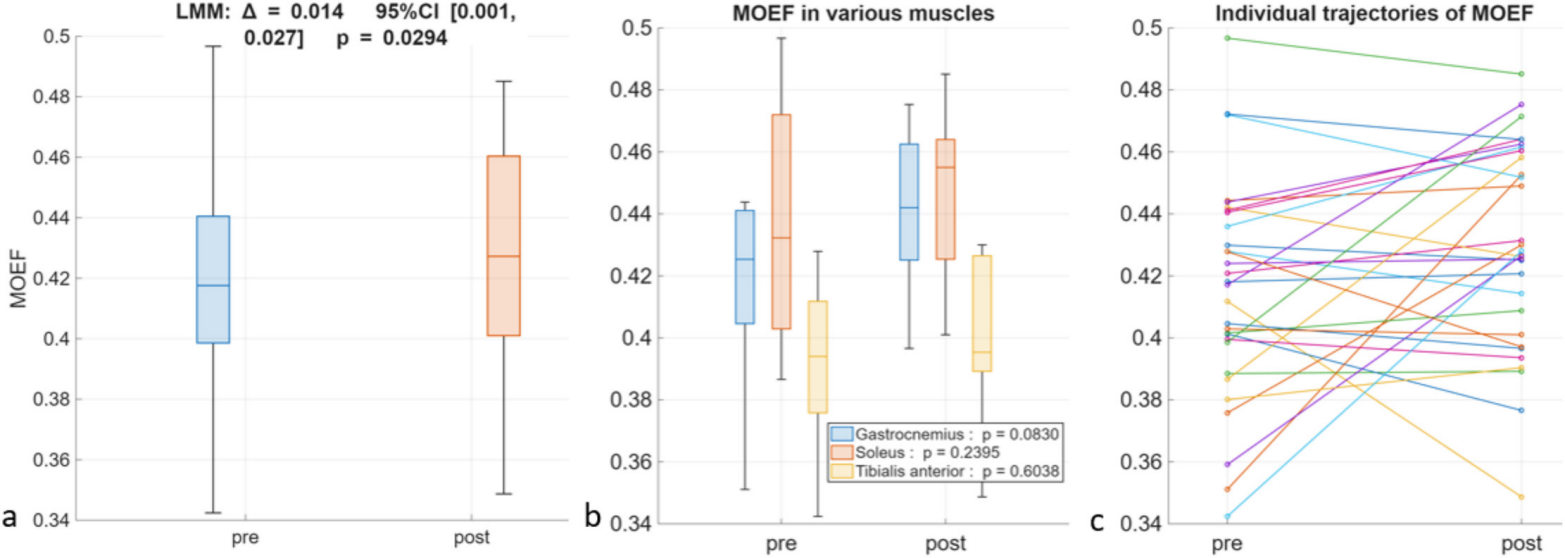
(a) MOEF increased significantly post‐exercise (*Δ* = 0.0142, 95% CI [0.0015, 0.0270], *p* = 0.029), with no significant interaction between time and muscle (*p* = 0.660). (b) Boxplots show group‐level distributions; paired *t*‐tests revealed no significant changes within individual muscles (all *p* > 0.05). (c) Spaghetti plots illustrate individual MOEF trajectories; each line represents the trajectory of an individual subject.

The main effect of time was also significant (*p* = 0.047), indicating a significant overall increase in MOEF following exercise. However, the time‐by‐muscle interaction was not significant (*p* = 0.660). The simplified model showed a modest but significant increase of *Δ* = 0.0142 (95% CI [0.0015, 0.0270], *p* = 0.029). No significant within‐muscle changes were detected (all *p* > 0.05), indicating a lack of significant differences in oxygen extraction responses among the individual muscles (Figure 5).

At baseline, SMVO_2_ differed significantly among the three calf muscles. One‐way repeated‐measures ANOVA revealed a significant main effect of muscle type (*F*(2, 18) = 18.80, *p* < 0.001). Mauchly's test indicated a violation of sphericity (W = 0.267, *p* = 0.005); therefore, the Greenhouse–Geisser correction was applied, which confirmed the significant effect (*p* = 0.001). Post hoc Bonferroni‐corrected comparisons showed that the gastrocnemius had markedly higher SMVO_2_ (72.38 ± 19.12 μmol O_2_/100 g/min) compared with both the soleus (34.14 ± 14.98 μmol O_2_/100 g/min, *p* = 0.003) and the tibialis anterior (37.95 ± 16.16 μmol O_2_/100 g/min, *p* = 0.009). No significant difference was found between the soleus and tibialis anterior (*p* = 0.571). After exercise (Figure 3, row f), SMVO_2_ increased across all muscles, with the most significant increase observed in the gastrocnemius, indicated by white circles (Figure 3).

**FIGURE 6:**
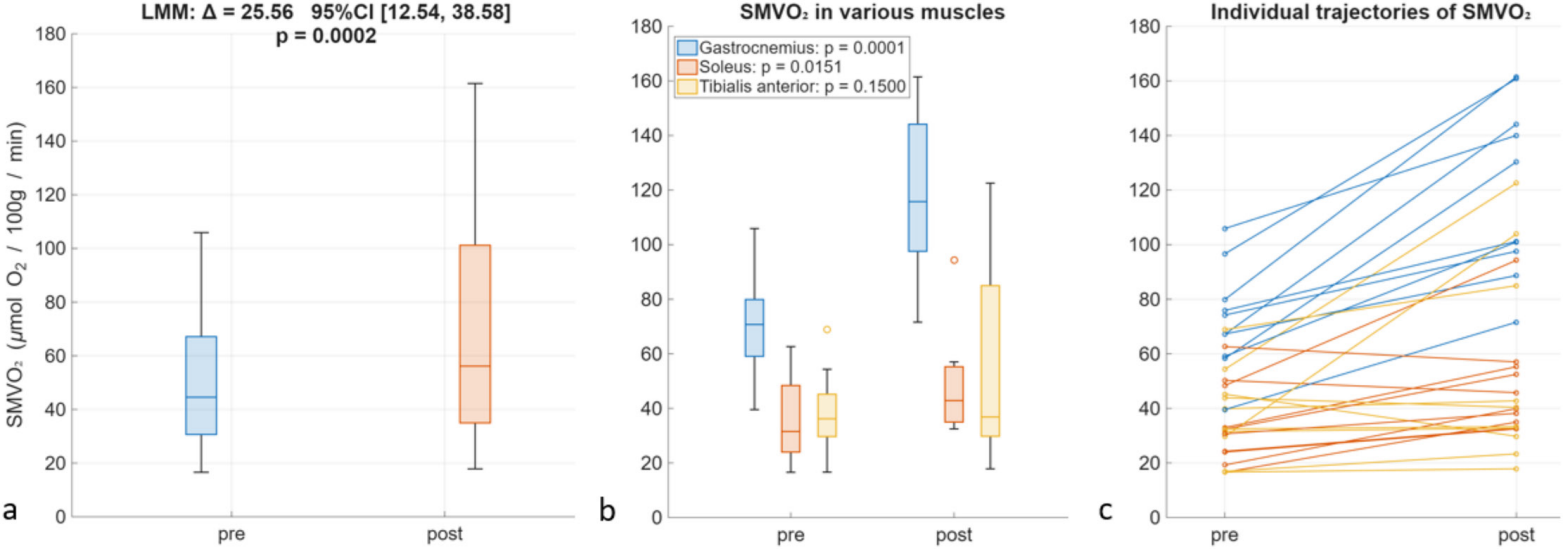
(a) SMVO_2_ increased significantly post‐exercise (*Δ* = 25.56, 95% CI [12.54, 38.58], *p* < 0.001), with a significant interaction between time and muscle (*p* = 0.028), indicating muscle‐specific responses. (b) Boxplots show significant increases in the gastrocnemius (*p* < 0.001) and soleus (*p* = 0.015), but not in the tibialis anterior (*p* = 0.150). (c) Spaghetti plots demonstrate consistent SMVO_2_ increases across most participants; each line represents the trajectory of an individual subject.

SMVO_2_ exhibited both a significant main effect of time (*p* < 0.001) and a significant interaction between time and muscle (*p* = 0.028), indicating heterogeneous metabolic responses among the calf muscles. Overall, SMVO_2_ showed an increase of *Δ* = 25.56 μmol O_2_/100 g/min (95% CI [12.54, 38.58], *p* < 0.001). The gastrocnemius showed the greatest relative increase in SMVO_2_ (68.8% ± 36.1%, *p* < 0.001), compared to the soleus (51.94% ± 44.02%, *p* = 0.015) and tibialis anterior (41.54% ± 84.52%, *p* = 0.150; Figure 6).

## 4 Discussion

Quantitative assessment of skeletal muscle perfusion and oxygenation provides key physiological insights, particularly in the field of exercise and microvascular function. The EE model [40] in this study simulates eccentric loading on calf muscles, as observed in athletic training and daily physical activity. It is also widely used in athletic conditioning and rehabilitation programs [45, 46]. The EE model could effectively increase oxygen metabolism in regional muscles without causing significant systemic fatigue [47]. Moreover, it offers a sustained time window for observing post‐exercise metabolic changes in muscle tissue [48].

In our study, VSASL was used to obtain MBF values. In ASL, pseudo‐continuous ASL (PCASL) is the most widely recommended approach [49]. However, spatially selective ASL suffers from label attenuation as blood travels from large arteries to the microvasculature, leading to signal loss—particularly in conditions with prolonged arterial transit time such as acute ischemic stroke, moyamoya disease, or arterial stenosis [50]. Velocity‐selective labeling overcomes this limitation by labeling blood within or near the imaging volume itself. By encoding slower velocities, VSASL minimizes arterial transit delays and is especially useful in organs outside the brain, where transit time is longer [20]. The calf is one of the most distal regions from the heart. Blood takes longer to reach the calf, especially in supine position or in older adults, due to reduced perfusion pressure and slower flow [51, 52]. In this study, VSASL was applied to quantitatively assess MBF in the calf muscles of healthy volunteers, aiming to minimize the influence of arterial transit delays. This technique enabled the acquisition of perfusion‐weighted images with sufficient SNR and spatial resolution, facilitating the evaluation of hypoperfusion status. Since VSASL is insensitive to transmission time, the signal loss caused by delay is small, so we obtained more calf muscle hypoperfusion areas information. At rest, the MBF values of gastrocnemius, soleus and tibialis anterior muscles are consistent with previous studies, where typical resting MBF in calf muscles measured by ASL has been reported in the range of 8–30 mL/100 g/min [53, 54]. However, the MBF values of the gastrocnemius muscles are significantly higher than those of the soleus and tibialis anterior muscles. This higher resting perfusion in the gastrocnemius may reflect its greater basal metabolic demand, likely related to its role in postural maintenance and readiness for movement [55]. In contrast, the soleus muscles showed the lowest resting MBF, which may be attributed to their predominantly slow‐twitch fiber composition and specialization in sustained, low‐intensity contractions [56]. Our results showed that MBF increase across all three muscles was 48.9%, particularly the gastrocnemius muscles with an increase of 60.4%. After EE, our MBF values were lower than those (6‐ to 9‐fold) reported in previous study using traditional ASL [30], likely due to differences in exercise models and scanning time points, as there was a 2‐ to 3‐min delay for scan preparation after EE. However, our results align with the expected perfusion response to EE and demonstrate good image quality. This regional difference reflects the selective activation of posterior calf muscles by the EE protocol used in this study. These findings support the ability of VSASL in capturing physiological changes and spatial differences in perfusion.

In our study, the ANN‐based GESSE was used to obtain MOEF images before and after EE. Our results demonstrate a modest but statistically significant overall increase in MOEF across the three muscles after EE, suggesting a sustained elevation in oxygen extraction following EE. However, no significant interaction between muscle type and time was observed. These findings suggest that while oxygen extraction increases systemically in response to exercise, the regional heterogeneity in MOEF responses is less pronounced than that observed for MBF. This may be explained by the nature of eccentric contractions, which require fewer motor units and thus consume less oxygen than concentric or isometric contractions [57, 58]. Prior work has demonstrated that ANN‐based reconstruction offers several advantages over conventional LSR, including reduced sensitivity to initial values, faster inference time, and better generalizability to noisy data, with feasibility and robustness established in brain MRI studies [35, 59]. The current framework was trained using simulated qBOLD signal curves to broadly cover heterogeneous qBOLD signal behaviors expected in skeletal muscle, where susceptibility effects may be influenced by vascular orientation, oxygen delivery‐consumption coupling, fat infiltration, and variable microvascular structure [1, 9, 30]. Therefore, the broad training ranges were used to improve fitting robustness across diverse signal conditions.

In the present study, baseline MOEF values in the gastrocnemius muscle (~0.42) were slightly higher than some PET‐ and NIRS‐based reports but remained broadly comparable to previously reported susceptibility‐based skeletal muscle MRI measurements, where resting gastrocnemius MOEF values of approximately 0.38–0.41 have been described [30]. Nevertheless, the current framework does not explicitly model skeletal muscle‐specific vascular architecture, and therefore the present study should be interpreted primarily as a feasibility application.

While %HbO_2_ from qBOLD MRI and tissue oxygen saturation from NIRS provide insights into oxygenation, both have key limitations. MRI‐based %HbO_2_ primarily reflects venous oxygen metabolism [36], whereas NIRS yields superficial, mixed arterial–venous signals with limited depth and potential contamination from skin and subcutaneous fat [1]. In contrast, SMVO_2_ enables deep regional mapping of oxygen consumption‐related changes, which is not achievable with surface‐limited optical methods. As such, SMVO_2_ may serve as a physiologically meaningful biomarker for differentiating between impaired oxygen delivery and impaired oxygen utilization, a distinction that cannot be made with conventional vascular imaging alone. Compared with conventional ASL‐qBOLD approaches, our VSASL and ANN‐based GESSE framework may provide enhanced sensitivity to microvascular oxygen extraction, and the measurement regions, acquisition timing, and more complex modeling assumptions likely also contribute to the observed differences.

However, SMVO_2_ calculation in the present study assumed a fixed arterial oxygen content ([O_2_] = 7.99 μmol/mL), as previously described [30] without subject‐specific hemoglobin measurements. Therefore, intersubject variations in hemoglobin concentration may influence the absolute SMVO_2_ values. Furthermore, qBOLD‐derived MOEF reflects susceptibility‐related deoxyhemoglobin signal behavior at the voxel level and may not be fully equivalent to the physiological OEF assumed in the classical Fick principle. Therefore, the calculated SMVO_2_ values should be interpreted primarily as relative oxygen consumption‐related measurements rather than absolute physiological quantification.

Furthermore, our SMVO_2_ results show that oxygen consumption in the gastrocnemius muscle is higher than in the other muscles at rest. This may reflect the differences in fiber composition and metabolic profiles among these muscles, which is consistent with our expectations. This study has implemented an approximate 2‐min delay after exercise to allow for scan preparation before the MRI acquisition begins. Although this delay may have reduced peak observable metabolic changes, it reflects a more practical approach for clinical implementation and may better capture the post‐exercise recovery window relevant for functional assessments and training adaptations. In addition, MBF and MOEF were acquired sequentially rather than simultaneously during the post‐exercise recovery period. Because both BF and OEF may change dynamically after exercise, the calculated SMVO_2_ values likely reflect different physiological time points, potentially leading to underestimation of peak post‐exercise SMVO_2_ responses. To reduce systematic bias related to acquisition order, participants were randomly assigned to different acquisition sequences, with five volunteers undergoing VSASL before GESSE and the remaining five undergoing GESSE before VSASL.

Our study has several limitations. First, the sample size was relatively small, which may limit the generalizability of the findings and increase intersubject variability in some measures. Moreover, the EE protocol was not normalized to individual maximal voluntary contraction, which may have introduced additional variability in exercise load across participants. Differences in daily habits among volunteers, such as regular hiking, cycling, or other physical activities may also contribute to intersubject variability in muscle metabolism. Second, only healthy young volunteers were included, so the applicability of this noncontrast MRI approach to older adults or clinical populations (e.g., patients with vascular or metabolic disorders) remains to be established. Furthermore, no independent reference standard was available for formal validation of absolute MOEF or SMVO_2_ measurements. In addition, test–retest reproducibility of the proposed framework was not evaluated in the current study. Therefore, the reproducibility and within‐subject variability of MOEF and SMVO_2_ measurements remain to be established in future studies.

## 5 Conclusion

This study demonstrates the feasibility of a fully noncontrast MRI framework combining VSASL and ANN‐based GESSE for quantitative, spatially resolved assessment of skeletal muscle perfusion and oxygen metabolism. The approach enables noncontrast evaluation of MBF, oxygen extraction, and oxygen consumption‐related changes while capturing muscle‐specific responses to EE.

## Author Contributions

All authors made substantial contributions to the conception or design of the work; or the acquisition, analysis, or interpretation of data for the work; All authors drafted the work or revised it critically for important intellectual content; All authors gave their final approval of the version to be published; All authors agree to be accountable for all aspects of the work in ensuring that questions related to the accuracy or integrity of any part of the work are appropriately investigated and resolved.

## Funding

The authors have nothing to report.

## Conflicts of Interest

The authors declare no conflicts of interest.

## Data Availability

The data that support the findings of this study are available on request from the corresponding author. The data are not publicly available due to privacy or ethical restrictions.
